# A novel diagnostic technology rapidly detects the presence of infection and defines antimicrobial sensitivities in organisms typical for joint infection

**DOI:** 10.1302/2046-3758.158.BJR-2025-0581.R1

**Published:** 2026-08-14

**Authors:** Isabelle L. Cochrane, Roselline Gabriella Gunarto, Frank Alexander Schildberg, Dieter Christian Wirtz, Robert J. H. Hammond, Phillip J. Walmsley

**Affiliations:** 1 School of Medicine, University of St Andrews, St Andrews, UK; 2 Department of Orthopaedics and Trauma Surgery, University Hospital Bonn, Bonn, Germany

**Keywords:** Septic arthritis, Periprosthetic joint infection, Antimicrobial resistance, Antimicrobial sensitivities, Rapid diagnostics, joint infections, Infection, Organism, antibiotics, methicillin-resistant Staphylococcus aureus (MRSA), Staphylococcus aureus, rifampicin, vancomycin, gentamicin, bacteria

## Abstract

**Aims:**

Infective arthritis is rare, but serious. Timely treatment with appropriate antimicrobial therapy is potentially life- and limb-saving, but our ability to deliver this is limited by current laboratory practices, which confer a delay of several days before antimicrobial sensitivities are available. We propose a novel diagnostic modality, the Scattered Light Integrating Collector (SLIC), which monitors bacterial growth in real time, allowing a diagnosis of infection to be made in minutes, and antimicrobial sensitivities to be delivered in under an hour.

**Methods:**

We collated data previously collected on SLIC relating to laboratory strains of *Staphylococcus aureus 25923* and *methicillin-resistant S. aureus* (MRSA) *29213*. Liquid cultures of the two organisms, with varying dilutions of target antibiotics, were compared in SLIC against positive (no antibiotic) and negative (no bacteria) controls. Three key measurements were derived: time to positivity (TTP - the timepoint at which significant bacterial growth was confirmed), time to sensitivities (TTS - the timepoint of statistically significant growth inhibition conferred by antibiotics vs the positive control), and IC50 (50% growth suppression relative to control).

**Results:**

TTP for *S. aureus* and MRSA was two minutes and 2.2 minutes, respectively. *S. aureus* was sensitive to gentamicin, with a minimum TTS of one minute, and IC50 achieved in under 19 minutes for all doses. Similarly, *S. aureus* susceptibility to vancomycin was established, with a minimum TTS of three minutes, and IC50 achieved in under 55 minutes for all but the lowest dose. MRSA was sensitive to rifampicin, with IC50 achieved in 16.5 minutes. Despite all tested antibiotics achieving TTS in under 30 minutes, only rifampicin achieved the criterion IC50 to declare susceptibility.

**Conclusion:**

SLIC rapidly determines the presence of infection and characterizes antimicrobial susceptibilities in organisms typical for joint infection.

Cite this article: *Bone Joint Res* 2026;15(8):1005–1016.

## Article focus

- Native and periprosthetic joint infections.
- The Scattered Light Integrating Collector as a novel diagnostic modality for infection.
- Rapid detection and sensitivity testing of *Staphylococcus aureus* and methicillin-resistant *S. aureus*.

## Key messages

- Infected joints present a diagnostic and therapeutic challenge.
- The Scattered Light Integrating Collector (SLIC) offers a rapid diagnostic for the presence of infection and inform clinical decisions around antibiotic choice.
- This approach would expedite the initiation of definitive treatment in the management of infected joints.

## Strengths and limitations

- This is a proof-of-concept of a novel diagnostic technology.
- Early results in organisms typical for joint infection align well with previous work done on infected blood cultures.
- Further work is required to validate standard operating procedure.

## Introduction

Infection of synovial joints comprises two distinct pathophysiological entities: native joint infections (septic arthritis (SA): incidence 4 to 10/100,000, mortality 11%),^1^ and periprosthetic joint infections (PJIs: incidence 1% of arthroplasty cases, 11% ten-year mortality.^2,3^ Both occur either via haematogenous spread from a remote primary infective source, or via direct inoculation.^4-6^ In SA, bacterial infection, toxin release, and the concomitant host immune response leads to rapid progressive destruction of joint tissues, meaning that even if the patient survives the acute episode, there can be sequelae including residual disability, ongoing chronic infection, and progressive joint failure.^4,5,7^ In PJI, this process is further complicated by the presence of prosthetic material, allowing biofilm formation and preventing successful treatment of infection by antibiotic therapy.^8^ Importantly, in all cases of infective arthritis, a widespread systemic response can develop, leading to a septic patient whose best chance of recovery is with reduction of the infective burden, in the form of a surgical washout, and early targeted antibiotic therapy.^5,9-11^

Detecting an organism and defining antimicrobial susceptibilities can routinely take 72 hours; this is longer in the case of fastidious organisms, and where infection has been partially suppressed by systemic antibiotics prior to sampling.^12,13^ Though empirical broad-spectrum antibiotics are often initiated in the interim, this represents poor antimicrobial stewardship,^14^ and due to the growing problem of antimicrobial resistance, the risk of treatment failure is high.^15,16^

Costs of treatment are also considerable, exceeding £30,000 for revising an infected total knee arthroplasty, and £20,000 for an infected total hip arthroplasty, with treatment success contingent on correct organism identification and appropriate antimicrobial therapy.^14,17-19^

**Fig. 1:**
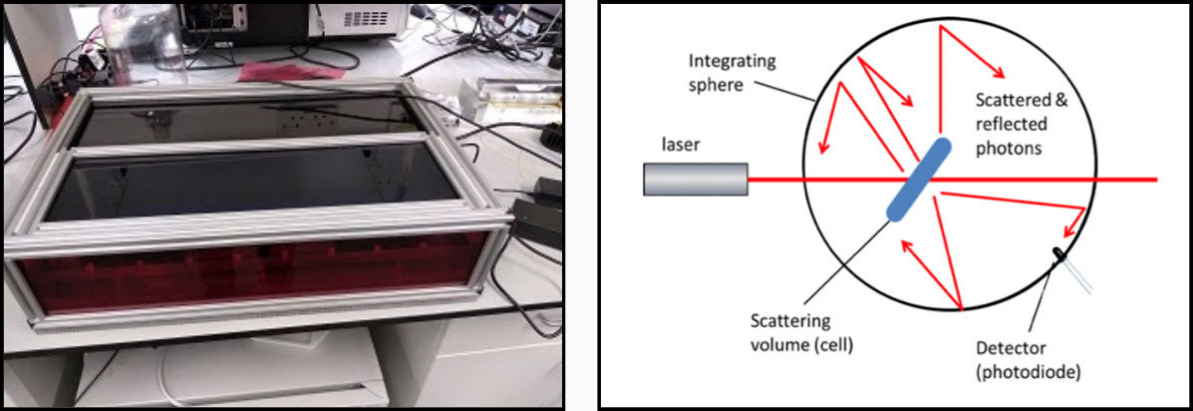
Left: the Scattered Light Integrating Collector (SLIC). Right: a diagrammatic representation of how SLIC quantifies bacteria in suspension. Figure reproduced from Hammond et al,^20^ with kind permission from the authors.

In response to these challenges, we propose a novel diagnostic technology, the Scattered Light Integrating Collector (SLIC). SLIC is a simple, cheap, label-free method for detecting and quantifying organism growth. The device works by passing a 658 nm red laser light through suspension of infective material, and collecting the scatter pattern as a signal via a photodetector (Figure 1). SLIC is able to detect organisms with a sensitivity of 100 colony-forming units (CFU)/ml, which is roughly 1,000 times more sensitive than current laboratory methods. This allows the device to track real-time changes in bacterial growth.^20^

SLIC has already been validated in clinical blood culture specimens infected with gram negative organisms, demonstrating 95.5% agreement with the current standard of care, in terms of categorizing organisms as either ‘sensitive’ or ‘resistant’ to an antibiotic. SLIC makes antimicrobial sensitivities available in under two hours, compared with the current laboratory standard of 10.5 hours from first pathogen detection.^21,22^ Similarly, in the context of urinary tract infections (UTIs), SLIC has a sensitivity of 86% and a specificity of 98% for UTI detection; susceptibility testing gives 94% agreement with current methods, and is deliverable within three hours.^23^

Given the success of SLIC in these clinical contexts, we now interrogate its applicability in infective arthritis. Early pilot testing of SLIC has demonstrated its ability to successfully detect and produce antimicrobial susceptibility profiles for a number of causative organisms in joint infections, including coagulase-positive and -negative staphylococci, and enterobacteriaceae.^24^

Our study focuses on *Staphylococcus aureus* and methicillin-resistant *S. aureus* (MRSA), as the two most prevalent organisms in infective arthritis.^5,10,25^ The primary aim is to determine how rapidly SLIC can detect these organisms, and the speed with which antimicrobial sensitivity testing can be carried out. The secondary aim is to explore a simple mathematical construct for deriving time to growth detection and for quantifying antimicrobial susceptibility.

## Methods

The data presented here represents a synthesis of previous results acquired using SLIC. We outline how the original data was acquired, and how it was processed to derive the present dataset.

### Bacterial selection and preparation

Two laboratory strains were used (*S. aureus* ATCC 25922, MRSA ATCC 29213), to ensure standardized properties and repeatability.

Laboratory stocks were defrosted from storage at -70°C, inoculated into Muller Hinton Cation Adjusted Broth medium - MHB (Sigma-Aldrich, USA), and allowed to grow in a 37°C incubator overnight.

### Sample preparation

Solutions were made up in UV-Vis cuvettes (Brand, UK) as follows: 1780 µL MHB, 20 µl bacteria at a concentration of 10^7^ colony forming units (CFU)/ml, and 200 µl of antibiotic solution at 10 × the target concentration (as determined by EUCAST minimum inhibitory concentration (MIC) breakpoints). This achieved a final volume of 2,000 µl, with a bacterial concentration of 10^5^ CFU/ml, and a final antimicrobial concentration in the same order of magnitude as the MIC.

For positive controls, 200 µl of sterile phosphate-buffered saline (PBS) or water was added, instead of the antibiotic solution. For negative controls, the bacterial solution was replaced with sterile media.

**Fig. 2:**
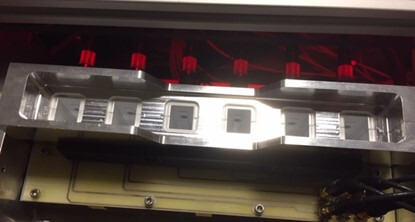
Samples are inserted into the Scattered Light Integrating Collector in individual cuvettes, each of which sits in one of the six wells, as depicted. During measurement, a 600 nm red laser light is directed at each of the six wells, allowing for individual measurements from each sample.

The cuvettes were inserted into the corresponding individual compartments within the SLIC device (Figure 2), and the temperature was equilibrated to 37°C. Scatter data were collected at one-second intervals for a minimum of 30 minutes.

### Retrospective acquisition of data

Overall, 129 antimicrobial sensitivity testing (AST) experiments carried out in SLIC from 20 February 2018 to 22 May 2023 were screened for data pertaining to *S. aureus* 25,923 and MRSA 29213. Experiments with the following characteristics were excluded: positive controls indicating abnormal growth, or negative controls indicating contamination; test duration < 30 minutes; antibiotic serial dilutions which did not include the MIC; antibiotics not used in clinical practice. A total of 21 AST experiments on *S. aureus* 25,923 and one AST experiment on MRSA 29213 were included in the final analysis. Each experiment consisted of at least triplicate technical repeats of the run, to improve accuracy and allow error margins to be calculated. Repeats were averaged prior to any further statistical analysis.

### Data processing and statistical analysis

Data processing and statistical analysis was carried out in Microsoft Excel 2016 (Microsoft, USA).

Raw SLIC output in decibels (dB) was converted to millivolts (mV) via the following formula:

$$mV=\left(1.0017\times e^{0.1151dB}\right)\times 4.2$$

Scatter data were normalized to the scatter level at time 0 s, to account for instrument offset in each channel. XY scatter plots were used to portray signal over time, with the data formatted as a moving-average time series (over a window of 60 seconds) to minimize the effect of background noise. 95% CIs of each 60-second window were calculated as ± 2 standard errors of the mean (SEM), and graphically represented as error bars. Differences between the antibiotic traces and the positive control for each organism were compared using a one-tail independent-samples *t*-test, with p < 0.05 as the limit for statistical significance.

Three key measurements were obtained. The first was time to positivity (TTP), which is the earliest time at which the presence of growing bacteria can be determined.

**Fig. 3:**
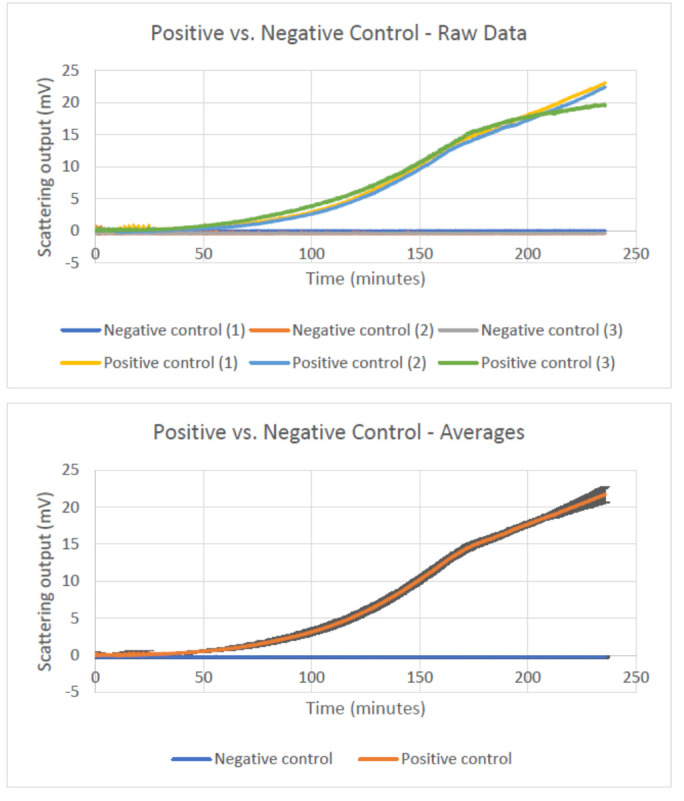
Top: the three rising traces represent ‘positive control’ data, in which bacteria (in this case *Staphylococcus aureus* 25923) are grown without antibiotic. The flat traces at the bottom of the graph are ‘negative control’ data, in which media only (without bacterial inoculation) is tested. Bottom: the rising trace represents the mean (standard error of the mean (SEM)) of the three positive control traces in the panel above. The flat trace overlying the x-axis represents the mean (SEM) of the negative control traces from the panel above.

TTP was defined as the timepoint at which the mean (95% CI) for the positive control for each organism (in normalized mV) was greater than, and remained above, 0 mV. 0 mV was selected, as this is the mean value of the negative control traces analyzed here (Figure 3).

The second key measurement was time to sensitivities (TTS), which is the earliest time at which antimicrobial sensitivities can be determined.

TTS was defined as the timepoint at which the mean value for a sample containing antibiotic became significantly different from the mean value of the positive control, and remained so going forward, indicating successful and ongoing growth suppression by the antibiotic.

The third and final key measurement was half maximal inhibitory concentration (IC50), which is a measure of the potency of an antibiotic against each organism.

To derive this measure, growth suppression was first calculated for each timepoint (t):

$$Growth\;Suppression\left(t\right)\left(\%\right)=\left(1-\frac{Signal\left(t\right)mV\left(antibiotictrace\right)}{Signal\left(t\right)mV\left(positivecontrol\right)}\right)*100$$

The lowest antibiotic dose at which sustained ≥ 50% growth suppression was achieved was defined as the IC50 for the organism in question. The organism was deemed susceptible to doses ≥ IC50 for a particular antibiotic.

TTP is used as a standalone measure, whereas TTS and IC50 are used in tandem as a two-pass system for determining sensitivity: once TTS has detected a statistically significant difference in growth rates between antibiotic and control traces, then IC50 can be used to quantify the difference in growth, and hence determine susceptibility. If TTS is not reached, the lack of statistically significant differences in growth between traces means that susceptibility cannot be declared.

### Determining antimicrobial susceptibilities

A key metric in AST is the minimum inhibitory concentration (MIC) for an antibiotic against a particular organism. This is the lowest dose of an antimicrobial agent, which when tested in a laboratory setting, results in a predetermined degree of inhibition of bacterial growth (often complete growth suppression). The MIC_(TEST)_ derived from a laboratory test for a given bacterial-antimicrobial combination can be compared to the MIC_(EUCAST)_ published in the EUCAST guidance tables. ^26^ If MIC_(TEST)_ ≤ MIC_(EUCAST)_, the organism is susceptible to the tested antibiotic.

We used two experimental designs to determine antimicrobial susceptibility.

In the experiments on *S. aureus*, doubling dilutions of each antibiotic were made, including the MIC_(EUCAST)_. IC50 was equated to MIC_(TEST)_. Therefore, if IC50 ≤ MIC_(EUCAST)_, the organism was deemed susceptible to the target antibiotic, otherwise it was deemed resistant.

In the experiments on MRSA, a panel of antibiotics was tested, each applied at its own MIC_(EUCAST)_. In cases where ≥ 50% growth suppression was achieved by an antibiotic, IC50 ≤ MIC_(EUCAST)_, and therefore the organism was deemed susceptible to the antibiotic.

## Results

### Bacterial growth curves

SLIC scatter output gives a characteristic shaped bacterial growth curve, with a clear lag phase, log phase, and stationary phase. This is clearly distinct from the flat trace of a negative control (Figure 3).

### Time to positivity

**Fig. 4:**
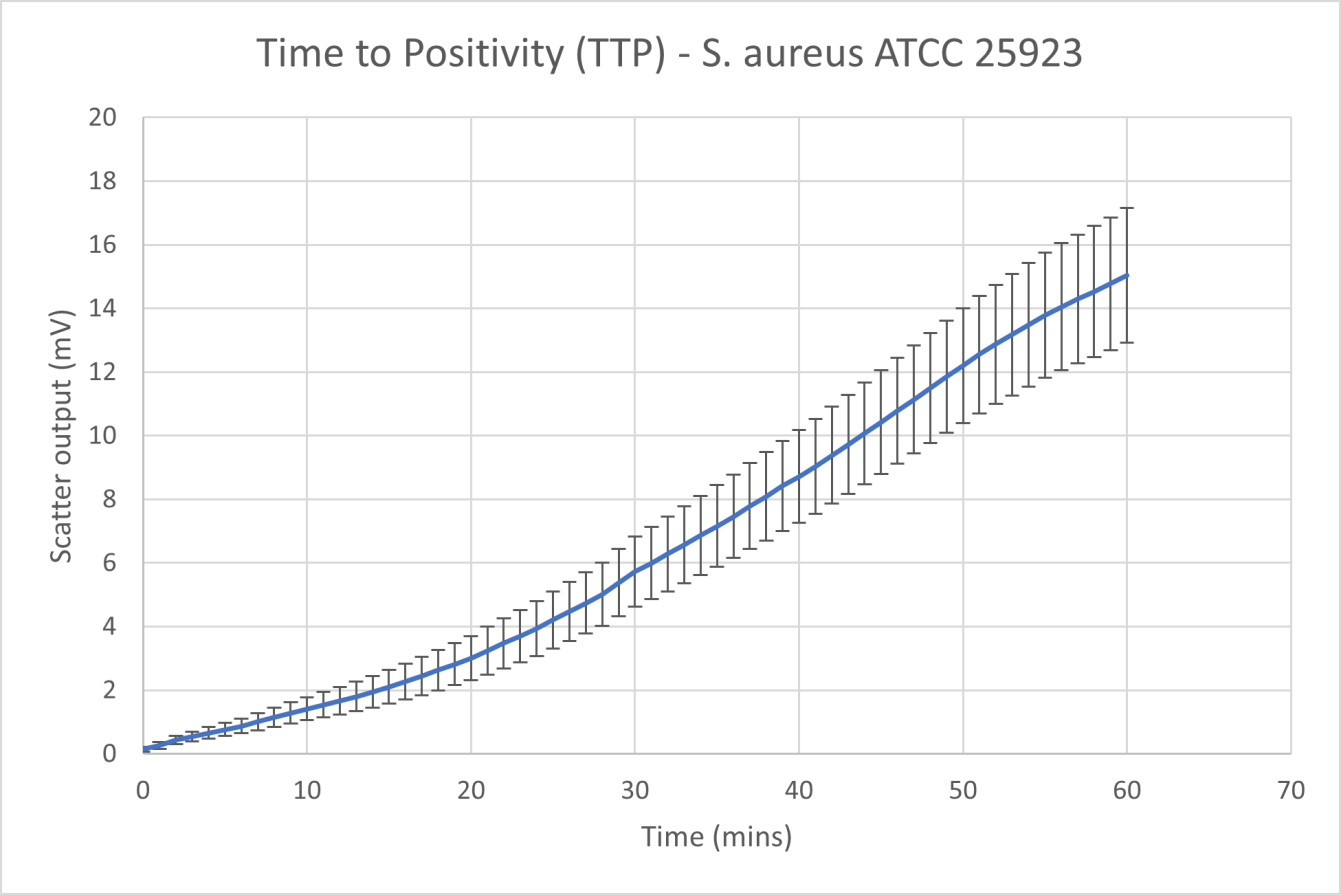
Collated moving-average scatter plot of ‘positive control’ samples containing *Staphylococcus aureus* 25923 without antibiotic. Time to positivity (TTP), with sustained growth above 0 mV, is achieved at 2.0 minutes.

The collated moving average derived from a total of 21 *S. aureus* 25,923 positive controls was used to derive TTP, which was 2.0 minutes (Figure 4).

**Fig. 5:**
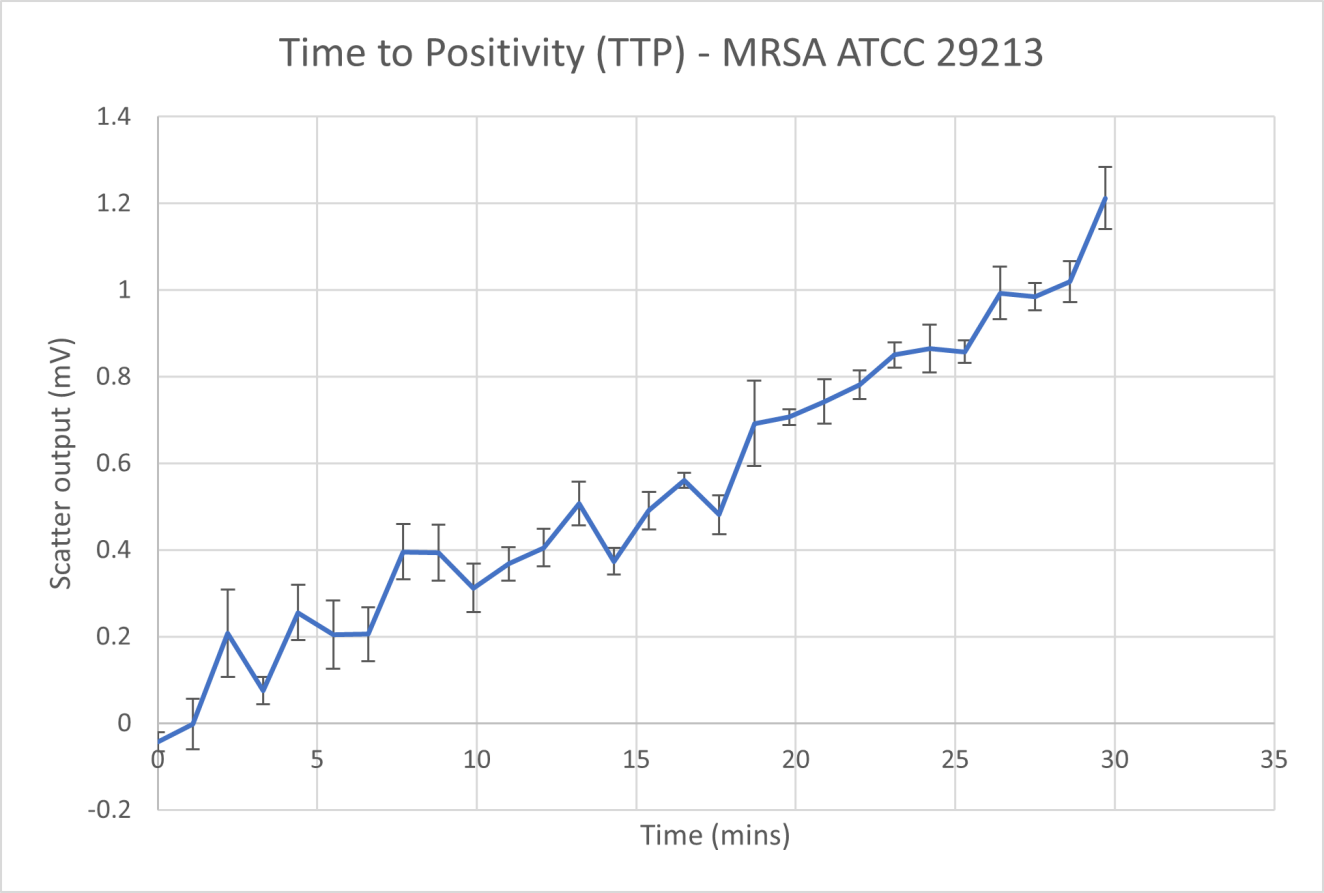
Collated moving-average scatter plot of ‘positive control’ samples containing MRSA 29213 without antibiotic. TTP, with sustained growth above 0 mV, is achieved at 2.2 minutes.

For MRSA 29213, a single dataset was available. The moving average of the control trace here indicated a TTP of 2.2 minutes (Figure 5).

### Antimicrobial sensitivity testing

**Fig. 6:**
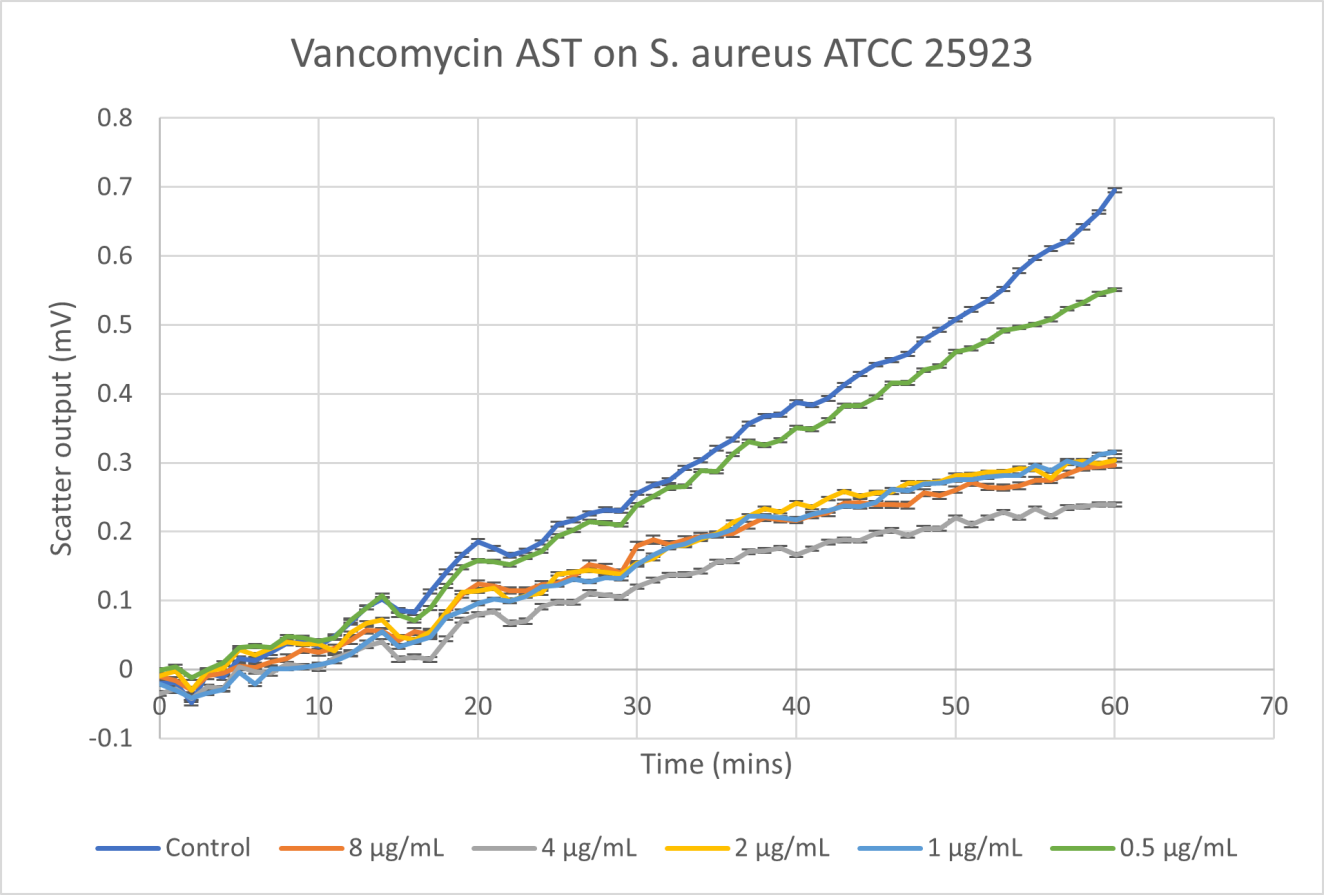
Collated moving-average scatter plot of vancomycin (0.5 to 8 μg/ml) tested against *Staphylococcus aureus* ATCC 25923 in the Scattered Light Integrating Collector, from 0 to 60 minutes, indicating statistically significant growth suppression (p < 0.05, independent-samples *t*-test) at all the tested antibiotic concentrations. AST, antimicrobial sensitivity testing.

Four datasets were available of *S. aureus* 25923 incubated with serial dilutions of vancomycin (0.5 to 8 µg/ml, EUCAST MIC = 2 µg/ml).^26^ At all but the lowest concentration, a statistically significant difference in bacterial growth was evident by 11 minutes, with statistical significance emerging at 15 minutes for 0.5 µg (Figure 6, Table I).

**Table I.** Table displaying the calculated independent-samples *t*-tests of moving average values taken per minute for vancomycin (0.5 to 8 μg/ml) tested against *Staphylococcus aureus* ATCC 25923.

Time (mins)	*t*-TEST 8	*t*-TEST 4	*t*-TEST 2	*t*-TEST 1	*t*-TEST 0.5
0	0.049601	0.00288	0.006775	0.445667	0.000148
1	0.335175	0.039424	0.00073	0.010378	1.34E-05
2	0.002496	0.171653	0.000384	0.086159	8.23E-12
3	0.254257	2.63E-06	0.283183	1.88E-10	0.159128
…					
10	0.0821	2.35E-10	0.235862	5.10E-09	0.033394
11	0.005195	2.33E-08	8.08E-06	1.94E-12	0.356485
…					
14	3.42E-14	3.56E-24	1.15E-11	1.65E-18	0.118126
15	8.86E-13	4.49E-29	2.19E-16	8.33E-25	0.042299
…					
60	2.33E-99	4.96E-119	2.54E-113	2.21E-115	6.35E-68

Each cell indicates the p-value generated from an independent-samples *t*-test comparing the antibiotic trace with the positive control trace at each recorded timepoint. A significant difference is defined as p-value ≤ 0.05 (5%), and in this case, the cell is highlighted in green. If the p > 0.05, the cell is highlighted in red. Where there is a lapse between illustrated timepoints (indicated by ellipses), this means that none of the traces have shown a sustained change from ‘not significant’ to ‘significant’ (or vice versa) within this time interval, and therefore the individual p-values for the intermediate timepoints are not shown. Time to sensitivities (TTS) is the timepoint which corresponds to the sustained change from ‘not significant’ to ‘significant’ for each trace. This is four minutes for 1 μg /ml and 4 μg /ml, one minute for 2 μg /ml and 8 μg /ml, and 15 minutes for 0.5 μg /ml. From TTS onwards, there is sufficient differentiation between the test and control traces, for conclusions about antimicrobial susceptibility to be drawn.

At least 50% growth inhibition was achieved at 52 minutes for the two highest concentrations of antibiotic, and at 105 minutes for the lowest concentration of vancomycin (Table II).

**Table II.** Table displaying the degree of *Staphylococcus aureus* ATCC 25923 growth suppression when exposed to each concentration of vancomycin (0.5 to -8 μg/ml), relative to the control group.

Time (mins)	IC50 8	IC50 4	IC50 2	IC50 1	IC50 0.5
32	34.0219	49.8177	35.1762	35.2369	3.7667
33	35.7631	53.0182	38.4966	37.8132	9.1686
…					
51	48.0217	59.732	45.8839	47.2559	10.753
52	50.545	58.7667	46.5276	47.8356	10.9312
53	52.2185	58.6176	48.0531	48.9586	10.8663
54	53.9193	61.9308	49.5101	51.3256	14.1787
55	54.1423	61.0042	51.3529	50.4324	16.2343
…					
104	84.0563	85.763	84.3194	81.8589	49.6587
105	84.5682	86.8704	85.0007	81.6102	50.307

Cells highlighted in red indicate < 50% of bacterial growth inhibition, whereas cells highlighted in green indicate ≥ 50% bacterial growth inhibition. Where there is a lapse between illustrated timepoints (indicated by ellipses), this means that none of the traces have shown a sustained change from ‘ < 50% growth inhibition’ to ‘ ≥ 50% growth inhibition’ (or vice versa) within this time interval, and therefore the individual growth suppression values for the intermediate timepoints are not shown.

This means that S. *aureus*, in this case, showed susceptibility to all but the lowest dose of vancomycin in under an hour, with extended testing revealing susceptibility to the lowest dose at a later timepoint.

As such, IC50 is 0.5 µg/ml, which falls below the EUCAST MIC,^26^ and therefore we infer this strain of *S. aureus* is sensitive to vancomycin.

**Fig. 7:**
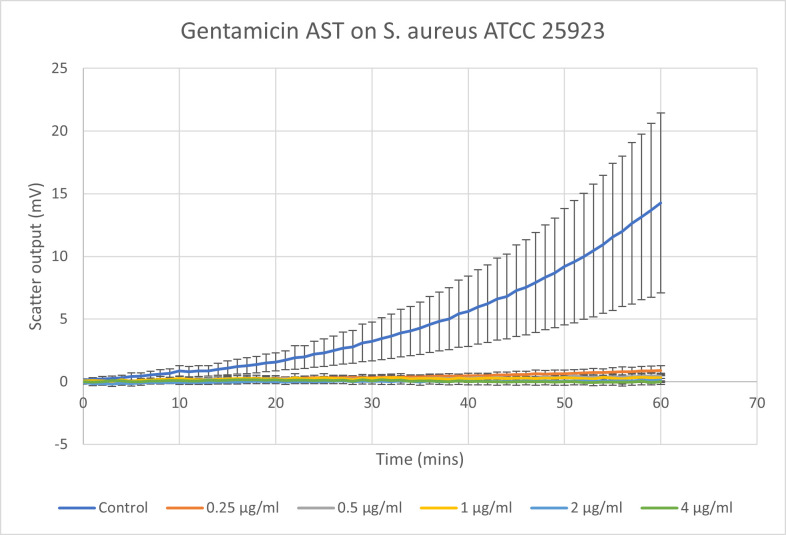
Collated moving-average scatter plot of gentamicin (0.25 to 4 μg/ml) tested against *Staphylococcus aureus* ATCC 25923 in the Scattered Light Integrating Collector device, from 0 to 60 minutes, indicating statistically significant growth suppression (p < 0.05, independent-samples *t*-test) at all the tested antibiotic concentrations. AST, antimicrobial sensitivity testing.

*S. aureus* + gentamicin: two datasets were available of S. *aureus* 25,923 incubated with serial dilutions of gentamicin (0.25 to 4 µg/ml, EUCAST MIC = 2 µg/ml).^26^ This antibiotic effected almost complete growth suppression at all doses tested (Figure 7), with all concentrations showing statistically significantly growth suppression relative to the control by 19 minutes (Table III).

**Table III.** Table displaying the calculated independent-samples *t*-tests of moving average values taken per minute for gentamicin (0.25 to 4 μg/ml) tested against *Staphylococcus aureus* ATCC 25923.

Time (mins)	*t*-TEST 0.25	*t*-TEST 0.5	*t*-TEST 1	*t*-TEST 2	*t*-TEST 4
0	0.166336	0.111014	0.373576	0.338235	0.13675
1	0.064261	0.04905	0.037534	0.330987	0.038986
2	0.123167	0.017285	0.118228	0.242586	0.029976
3	0.158728	0.203219	0.211317	0.264977	0.043065
4	0.034391	0.156111	0.075694	0.106224	0.02336
…					
8	0.026478	0.05193	0.021118	0.119057	0.013796
9	0.004348	0.003469	0.003537	0.045911	0.005648
…					
18	0.010486	0.012422	0.008083	0.06528	0.012025
19	0.018074	0.019046	0.012356	0.042106	0.012425

Each cell indicates the p-value generated from an independent-samples *t*-test comparing the antibiotic trace with the positive control trace at each recorded timepoint. A significant difference is defined as p-value ≤ 0.05 (5%), and in this case, the cell is highlighted in green. If the p > 0.05, the cell is highlighted in red. Where there is a lapse between illustrated timepoints (indicated by ellipses), this means that none of the traces have shown a sustained change from ‘not significant’ to ‘significant’ (or vice versa) within this time interval, and therefore the individual p-values for the intermediate timepoints are not shown. Time to sensitivities (TTS) is the timepoint which corresponds to the sustained change from ‘not significant’ to ‘significant’ for each trace. This is one minute for 4 μg /ml, four minutes for 0.4 μg /ml, eight minutes for 4 μg /ml, nine minutes for 0.5 μg /ml, and 19 minutes for 2 µg/ml. From TTS onwards, there is sufficient differentiation between the test and control traces, for conclusions about antimicrobial susceptibility to be drawn.

All concentrations showed ≥ 50% growth inhibition within eight minutes (Table IV).

**Table IV.** Table displaying the degree of *Staphylococcus aureus* ATCC 25923 growth suppression when exposed to each concentration of gentamicin (0.25 to -4 μg/ml), relative to the control group.

Time (mins)	IC50 0.25	IC50 0.5	IC50 1	IC50 2	IC50 4
0	148.025	263.192	85.0013	-153.184	167.533
1	86.6329	134.562	131.829	71.1119	162.356
2					
3	79.6153	74.6103	102.547	78.5899	149.394
4	89.4954	63.6097	111.16	90.3526	109.167
5	77.9855	78.1742	114.47	79.2252	114.504
6	72.7553	42.4281	97.4773	57.8017	110.195
7	61.8421	44.878	106.321	61.5105	92.7934
8	68.6152	71.8009	105.412	76.7759	105.173

Cells highlighted in red indicate < 50% of bacterial growth inhibition, whereas cells highlighted in green indicate ≥ 50% bacterial growth inhibition. Where there is a lapse between illustrated timepoints (indicated by ellipses), this means that none of the traces have shown a sustained change from ‘ < 50% growth inhibition’ to ‘≥ 50% growth inhibition’ (or vice versa) within this time interval, and therefore the individual growth suppression values for the intermediate timepoints are not shown. Timepoints > eight minutes not illustrated, as all traces consistently show ongoing ≥ 50% growth inhibition.

As above, IC50 ≤ MIC_(EUCAST)_,^26^ and therefore this strain of *S. aureus*, can be deemed sensitive to gentamicin.

Though the IC50 standard for growth inhibition is met as early as eight minutes, a robust result for sensitivities can only be declared from TTS as, by definition, this is the point at which the difference between the test and control traces is statistically significant.

**Fig. 8:**
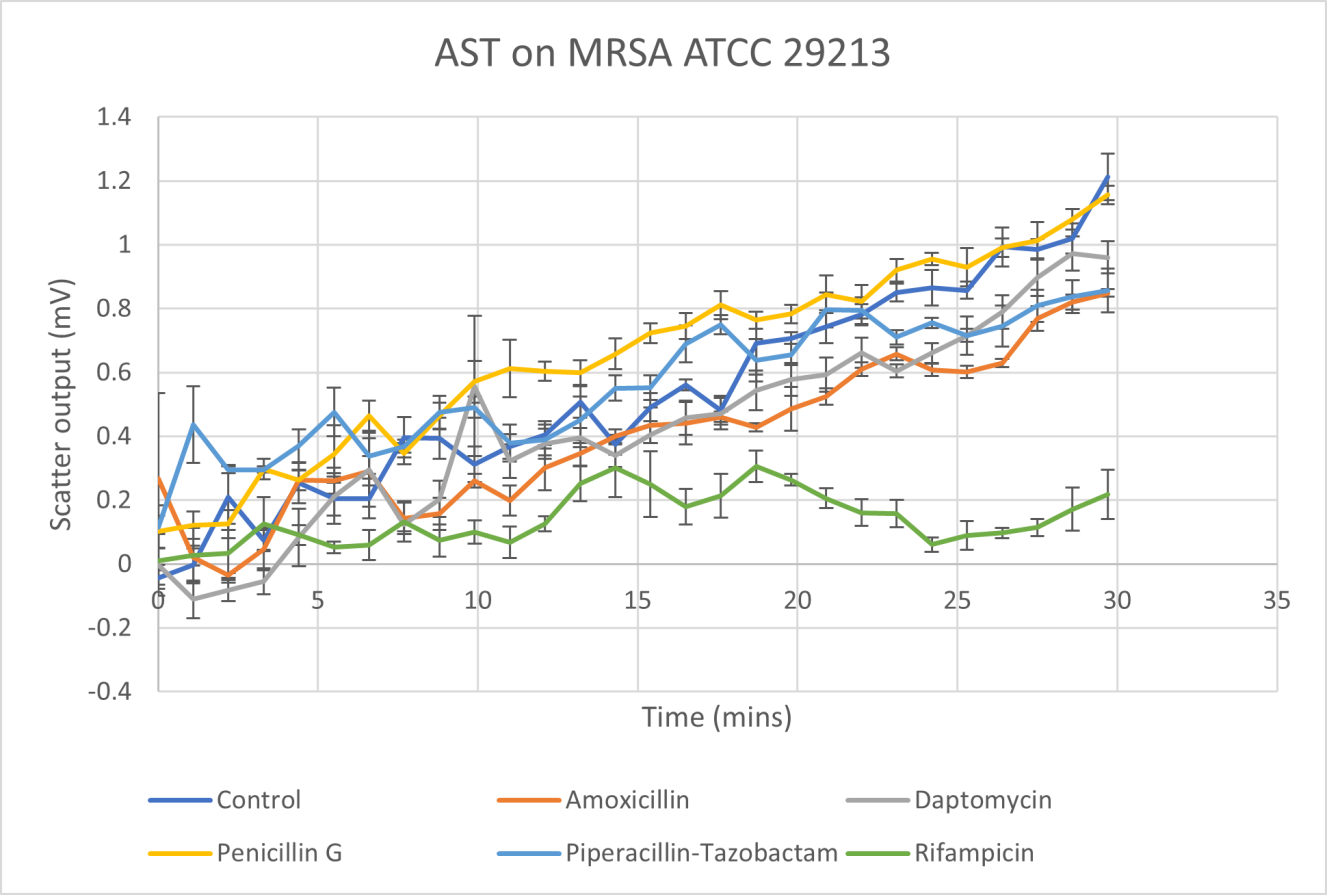
Collated moving-average scatter plot testing a panel of antibiotics (amoxicillin, daptomycin, penicillin G, piperacillin-tazobactam, rifampicin) against methicillin-resistant *Staphylococcus aureus* (MRSA) ATCC 29213 in the Scattered Light Integrating Collector device over the complete test duration. Rifampicin appears to be the only antimicrobial drug to be effective in bacterial inhibition. AST, antimicrobial sensitivity test.

To simulate an AST panel, a single dataset of MRSA 29213 incubated with a panel of five different antibiotics at their respective MICs was interrogated (Figure 8). For amoxicillin vs staphylococci, no EUCAST MIC was available (due to commonly encountered resistance), so the MIC for enterococci was used as a substitute.^26^

The traces were compared to a no-antibiotic positive control.

By 30 minutes, a sustained significant difference in growth was demonstrated in all antibiotics bar penicillin G, which at no point achieved a statistically significant suppression in growth relative to the control (Table V).

**Table V.** Table displaying the calculated independent-samples *t*-tests of moving average values taken per minute. ‘*t*-TEST amox’, ‘*t*-TEST dapto’, ‘*t*-TEST pen G’, ‘*t*-TEST piptaz’, and ‘*t*-TEST rifam’ columns indicate the calculations for amoxicillin, daptomycin, penicillin G, piperacillin-tazobactam, and rifampicin against methicillin-resistant *Staphylococcus aureus* 29213, respectively.

Time (mins)	*t*-TEST amox	*t*-TEST dapto	*t*-TEST pen G	*t*-TEST piptaz	*t*-TEST rifam
0	0.147864	0.349304	0.019237	0.033185	0.291629
…					
14.3	0.256773	0.248955	0.001318	0.006003	0.238373
15.4	0.226605	0.071803	0.002531	0.165869	0.037098
…					
17.6	0.338863	0.438341	0.000934	0.001278	0.008848
18.7	0.018462	0.123532	0.253849	0.332886	0.006391
…					
24.2	0.00234	0.009187	0.0858	0.052996	5.27E-06
25.3	0.000108	0.002648	0.158243	0.037462	3.14E-06
…					
28.6	0.00467	0.268296	0.170611	0.020974	2.51E-05
29.7	0.003757	0.022481	0.253757	0.011596	0.000359

Each cell indicates the p-value generated from an independent-samples *t*-test comparing the antibiotic trace with the positive control trace at each recorded timepoint. A significant difference is defined as p-value ≤ 0.05 (5%), and in this case, the cell is highlighted in green. If the p > 0.05, the cell is highlighted in red. Where there is a lapse between illustrated timepoints (indicated by ellipses), this means that none of the traces have shown a sustained change from ‘not significant’ to ‘significant’ (or vice versa) within this time interval, and therefore the individual p-values for the intermediate timepoints are not shown. Time to sensitivities (TTS) is the timepoint which corresponds to the sustained change from ‘not significant’ to ‘significant’ for each trace. This 15.4 minutes for rifampicin, 18.7 minutes for amoxicillin, 25.3 minutes for piperacillin-tazobactam, 29.7 minutes for daptomycin. Statistical significance for the penicillin G trace vs the control trace is never reached. From TTS onwards, there is sufficient differentiation between the test and control traces, for conclusions about antimicrobial susceptibility to be drawn. Where TTS is never reached (lack of statistically significant difference between traces), resistance is implied.

However, the only antibiotic which met the criterion of 50% growth suppression during the test was rifampicin, at 16.5 minutes (Table VI).

**Table VI.** Table displaying the proportion of MRSA 29213 growth present when exposed to different antibiotics against MRSA 29213. ‘IC50 amox’, ‘IC50 dapto’, ‘IC50 pen G’, ‘IC50 piptaz’, and ‘IC50 rifam’ columns indicate the calculations for amoxicillin, daptomycin, penicillin G, piperacillin-tazobactam, and rifampicin, respectively.

Percentage Bacterial Growth Inhibition (MRSA ATCC 29213)					
Time (min)	IC50 amox	IC50 dapto	IC50 pen G	IC50 piptaz	IC50 rifam
0	726.631	94.2975	339.8	373.369	125.691
…					
15.4	11.4489	17.4484	-47.2574	-12.5999	48.9279
16.5	21.4101	18.3814	-32.9191	-22.8844	68.0429
…					
29.7	29.9645	20.7791	4.6518	29.3789	82.0279

Cells highlighted in red indicate < 50% of bacterial growth inhibition, whereas cells highlighted in green indicate ≥ 50% bacterial growth inhibition. Where there is a lapse between illustrated timepoints (indicated by ellipses), this means that none of the traces have shown a sustained change from ‘ < 50% growth inhibition’ to ‘ ≥ 50% growth inhibition’ (or vice versa) within this time interval, and therefore the individual growth suppression values for the intermediate timepoints are not shown. Rifampicin is the only antibiotic on the panel that meets the IC50 criterion for sensitivity.

On this basis, of the tested antibiotics, rifampicin was the only one to which this MRSA strain was deemed susceptible.

## Discussion

Our results illustrate that SLIC can be used to rapidly identify the presence of *S. aureus* and MRSA, the two most common organisms implicated in PJI,^10,25^ in under three minutes. We were also able to establish antimicrobial sensitivities in under an hour for both organisms. The mathematical constructs underlying both detection and sensitivities are simple, intuitive, and easy to use, requiring minimal computational power.

These results are in concordance with previous work carried out on blood cultures and urine detection,^21,22^ and build on the emerging picture that SLIC has good applicability in organisms relevant to infective arthritis.^24^

SLIC is rapid, sensitive, and cheap (manufacturing cost approximately £11,000, with estimated running costs < £2.00/run), placing it in a uniquely strong position among other novel diagnostic methods for joint infections. The slow, labour-intensive nature of culture-based methods has been mitigated to some extent by the introduction of matrix-assisted laser desorption/ionization time-of-flight mass spectrometry in many centres. This allows organisms (and in some cases, resistance patterns) to be identified within five hours. However, this technology still relies on a 48-hour initial culture step, upfront costs are high, and the informatic-based identification of resistance patterns requires further clinical validation.^27,28^ Newer polymerase chain reaction (PCR)-based techniques are comparably fast to SLIC, and can deliver in under an hour, but are limited in the organisms they can detect by commercial primer panel selection.^29,30^ As SLIC detection is label-free, this limitation does not apply. Metagenomic next-generation sequencing (mNGS) is more versatile than PCR, capturing a broad range of organisms with high sensitivity; as with SLIC, these characteristics lend themselves to the work-up of culture-negative PJIs, which sit at a rate of 7% to 15%, and present a considerable diagnostic challenge.^13^ Nevertheless, mNGS startup and running costs are high, specialist laboratory setup is required, and the large computational resources required mean results can take 48 hours to be returned.^31,32^ Of note, molecular diagnostics provide information on genotypic resistance, which may not always correlate with the organism’s phenotypic resistance pattern,^33^ whereas, much like culture-based methods, SLIC provides direct information on the resistance phenotype. Another specific strength of SLIC is its ability to detect mycobacteria and fungi, without additional adaptations or special culture conditions being required.^34,35^

We acknowledge certain limitations in the present work. Our results are based on bacteria growing in media, a homogeneous, clear, non-viscous fluid with predictable, reproducible properties. However, synovial fluid is a viscous plasma ultrafiltrate which is rich in proteins (particularly hyaluronic acid), and whose properties vary across pathologies, including infection, with changes in viscosity, opacity, colour, and cell composition.^10,36^ These factors may affect the performance of an optical device, such as SLIC, potentially increasing noise and offset, and therefore compromising sensitivity and time to results. SLIC is equipped to handle low bacterial loads, the sample volume required is low, meaning synovial fluid in the final solution will be dilute. Should this still prove problematic however, there is a good precedent in the literature for successfully processing synovial fluid, making it more amenable to visual and spectroscopic analysis.^37,38^ Further research is required into SLIC’s performance in the face of polymicrobial specimens and highly resistant pathogens.

Looking ahead, we plan further validation of the mathematical methods used, tested against EUCAST standards. We will characterize the capabilities of SLIC in running patient samples directly, including synovial fluid, sonication fluid, and tissue samples. By expanding new iterations of SLIC to include more test wells (16-well version currently operational), exploring microfluidics-based approaches, and by migrating our current Microsoft Excel-based analysis to a more computationally robust system, we plan for upscaling, and improving sample throughput.

## Data Availability

The data that support the findings for this study are available to other researchers from the corresponding author upon reasonable request.
